# Vitamin D: Promises on the Horizon and Challenges Ahead for Fighting Pancreatic Cancer

**DOI:** 10.3390/cancers13112716

**Published:** 2021-05-31

**Authors:** Daoyan Wei, Liang Wang, Xiangsheng Zuo, Robert S. Bresalier

**Affiliations:** 1 Unit 1466, Department of Gastroenterology, Hepatology, and Nutrition, The University of Texas MD Anderson Cancer Center, 1515 Holcombe Boulevard, Houston, TX 77030, USA; lwang11@mdanderson.org; 2 Gastrointestinal Medical Oncology, The University of Texas MD Anderson Cancer Center, Houston, TX 77030, USA; xzuo@mdanderson.org

**Keywords:** vitamin D, pancreatic cancer, cancer risk, cancer survival, immunotherapy

## Abstract

**Simple Summary:**

Pancreatic cancer is an almost universally lethal cancer, largely due to its late diagnosis, early metastasis, and therapeutic resistance. This highlights the need to develop novel and effective intervention strategies to improve the outcomes of patients with pancreatic cancer. Vitamin D is one of the hottest topics in cancer research and clinics because of its pleiotropic functions on the hallmarks of cancer. Here we critically review past and current efforts that define the effects of vitamin D on the risk, incidence, patient survival, and mortality of pancreatic cancer. We also provide overviews on the opportunities and challenges associated with vitamin D as an economic adjunct to improve the efficacy of immunotherapy and chemo- or radiotherapy for pancreatic cancer.

**Abstract:**

Pancreatic cancer has a dismal prognosis, while its incidence is increasing. This is attributed, in part, to a profound desmoplastic and immunosuppressive tumor microenvironment associated with this cancer and resistance to current available therapies. Novel and effective intervention strategies are urgently needed to improve the outcomes of patients with pancreatic cancer. Vitamin D has pleiotropic functions beyond calcium–phosphate homeostasis and has been extensively studied both in the laboratory and clinic as a potential preventive agent or adjunct to standard therapies. Accumulating evidence from ecological, observational, and randomized controlled trials suggests that vitamin D has beneficial effects on risk, survival, and mortality in pancreatic cancer, although controversies still exist. Recent advances in demonstrating the important functions of vitamin D/vitamin D receptor (VDR) signaling in the regulation of stromal reprogramming, the microbiome, and immune response and the emergence of checkpoint immunotherapy provide opportunities for using vitamin D or its analogues as an adjunct for pancreatic cancer intervention. Many challenges lie ahead before the benefits of vitamin D can be fully realized in pancreatic cancer. These challenges include the need for randomized controlled trials of vitamin D to assess its impact on the risk and survival of pancreatic cancer, optimizing the timing and dosage of vitamin D or its analogues as an adjunct for pancreatic cancer intervention and elucidating the specific role of vitamin D/VDR signaling in the different stages of pancreatic cancer. Nevertheless, vitamin D holds great promise for reducing risk and improving outcomes of this disease.

## 1. Introduction 

Pancreatic cancer (PC) is currently the third-leading cause of cancer-related death in the United States and is predicted to be the second by 2030 [1,2]. PC has a dismal prognosis, with an overall survival from diagnosis of about 8 months, and a 5-year survival rate of less than 9%, which is largely due to its late diagnosis and resistance to conventional therapy. New treatments, such as checkpoint immunotherapy, which has achieved impressive success in several other malignancies, have had little efficacy in treating PC. This lack of efficacy is due in part to the unique, profoundly desmoplastic and immunosuppressive tumor microenvironment that prevents the infiltration and activation of effector T cells and their subsequent elimination of tumor cells [3]. Moreover, many patients develop adverse events that prevent them from continuing treatment with immune checkpoint inhibitors [4], and these inhibitors have led to a rapid increase in the cost of cancer care [5]. Thus, the development of novel and cost-effective intervention strategies is urgently needed to improve the outcomes of patients with PC. 

Scientists obtained evidence that the active form of vitamin D (1, 25-dihydroxy vitamin D_3_ [1, 25(OH)_2_D_3_], also called calcitriol) is a hormone that not only regulates calcium–phosphate homeostasis but also has pleiotropic effects on the regulation of cell proliferation and differentiation and on antimicrobial and immune responses [6,7]. Numerous epidemiological and clinical observational studies suggest that a higher intake of vitamin D is associated with a lower risk of cancer including PC [8,9,10,11], although a few studies contradict these findings [12], and vitamin D deficiency is prevalent among PC patients [13]. Higher levels of blood 25-hydroxyvitamin D [25(OH)D] are associated with longer survival duration in patients with PC [14]. Experimental evidence indicates that vitamin D exerts anticancer effects by inhibiting cancer cell proliferation, inducing apoptosis and differentiation and potentiating chemo- or radiotherapy in various cancers [15,16,17,18,19,20,21]. Interestingly, experimental data also demonstrate that vitamin D regulates the tumor microenvironment, particularly cancer-associated fibroblast reprogramming, to facilitate tumor repression [20,22]. Moreover, vitamin D was found to decrease the risk of colitis, a common side effect of checkpoint inhibitors [23]. 

While many challenges remain to be resolved, these lines of evidence suggest the promising use of vitamin D as an additional cost-effective agent for fighting PC.

## 2. Vitamin D Sources, Metabolism, and Signaling 

Vitamin D is the common name of a group of fat-soluble secosteroids with two major forms in humans, vitamin D_2_ (ergocalciferol) and vitamin D_3_ (cholecalciferol). Vitamin D_3_ is generated in the skin after exposure to ultraviolet B (UVB) light via a process that involves photolysis of cutaneous 7-dehydrocholesterol. Vitamin D is also found in some foods, D_3_ in animal sources and D_2_ in plant and fungi sources, or can be taken as a supplement. Dietary sources of vitamin D are biologically inert, however, and must undergo two hydroxylations for activation in the body. 

Carried by vitamin D binding proteins (VDBP) in the bloodstream, vitamin D reaches the liver, where it is hydroxylated by sterol 27-hydroxylase (CYP27A1) and converted to 25(OH)D, the most stable vitamin D metabolite with a serum circulation half-life of 15 days [24]. Thus, the serum concentration of 25(OH)D is used as a biomarker to determine the vitamin D status of a person [25]. There is a lack of consensus, however, regarding the optimal concentration of vitamin D in the blood. In the United States, an expert committee of the Food and Nutrition Board of the Institute of Medicine categorized the serum 25(OH)D levels as follows: deficiency, less than 30 nmol/L (12 ng/mL); inadequacy, between 30 nmol/L (12 ng/mL) and 50 nmol/L (20 ng/mL); normal, between 50 nmol/L (20 ng/mL) and 125 nmol/L (50 ng/mL); and high, higher than 125 nmol/L (50 ng/mL) [24]. The Endocrine Society defines the serum 25-(OH)D levels differently: deficiency, less than 20 ng/mL (50 nmol/L); insufficiency, 21–29 ng/mL (52 to 72 nmol/L) [26]. These cut-offs are based on the levels of parathyroid hormone and the activity of the intestinal calcium transporter that normalizes 25-(OH)D levels [27,28,29]. It should be noted that the total vitamin D concentration in blood is influenced by many factors, such as age, dietary habits, intestinal absorption capacity, liver and kidney functions, sex steroid levels (in particular estrogens), and genetic background [30,31]. Therefore, one should be cautious in defining the vitamin D status of a subject. 

25(OH)D is further activated in the kidneys by 1α-hydroxylase (CYP27B1) to 1,25(OH)_2_D, which is the biologically active form of vitamin D and the high-affinity ligand of the steroid hormone nuclear vitamin D receptor (VDR) transcription factor. 1,25(OH)_2_D is subsequently catabolized by CYP24A1 to their inactive forms 24,25(OH)_2_D and 1,24,25(OH)_3_D (or 1,23,25(OH)_3_D) [32]. The expression of CYP24A1 is highly induced by 1, 25(OH)_2_D, thus, forming a feed-back loop to limit vitamin D over-activation. 

**Figure 1:**
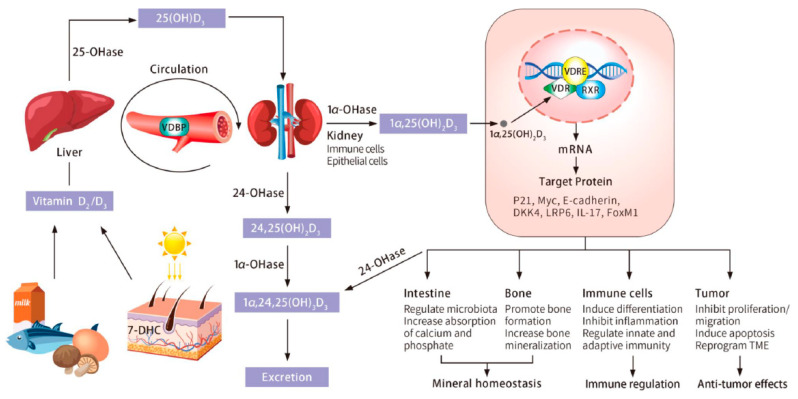
Vitamin D metabolism and biological functions. Vitamin D is obtained either from precursors in food or produced in the skin from conversion of 7-DHC through sunlight exposure. In the liver, vitamin D_3_ is hydroxylated to form 25(OH)D_3_. A second hydroxylation produces the active metabolite 1⍺,25(OH)_2_D_3_. This step mainly takes place in the kidney but also in other tissues, such as immune cells and epithelial cells expressing 1⍺-hydroxylase. Vitamin D metabolites are carried by VDBP in the blood circulation. 1⍺,25(OH)_2_D_3_ enters cells and binds to VDR, which enables the formation of VDR-RXR complex. This complex translocates into cell nuclei and binds to VDRE to regulate gene expression. Vitamin D has various biological functions in multiple organs and tissues. Active forms of vitamin D are degraded in the kidney as well as other target tissues and finally excreted by urine. 7DHC, 7-dehydrocholesterol; VDBP, vitamin D binding protein; VDR, vitamin D receptor; RXR, retinoid X receptor; VDRE, vitamin D response element.

A wide variety of cells including gut epithelia, immune cells, and cancer cells express both CYP27B1 and VDR, which provides the molecular basis for 1,25(OH)_2_D to exert its multifunctional role in the human body (Figure 1) [6,7]. 1,25(OH)_2_D ligand-activated VDR binds to more than 10,000 loci within the human genome, regulating the transcriptional expression of approximately 1000 target genes in many different cell types including epithelial cells, fibroblasts, and almost all the cells of the immune system [33,34]. Accumulating evidence shows that a low circulating 25(OH)D level is associated with an increased risk of developing several different diseases including those associated with chronic inflammation [35,36]. The connection of low serum 25(OH)D with chronic inflammation raised the question of whether low serum 25(OH)D is a cause or a consequence of chronic inflammation given the broad influence of vitamin D on immune cell functions [35,36,37]. Some experts now believe that low serum 25(OH)D is most likely an effect of chronic inflammation rather than the cause [36,38]; however, further studies are needed to answer this question [37]. Additionally, vitamin D deficiency is highly prevalent in patients with newly diagnosed cancer [39,40,41]. In order to maximize the beneficial effects of vitamin D for health, it is suggested to increase the intake of vitamin D and/or exposure of sunlight to maintain serum 25(OH)D at least at 30 ng/mL (75 nmol/L) and preferably at 40–60 ng/mL (100–150 nmol/L) [35,42,43,44]. 

Because excessive sun exposure can damage the skin and cause skin cancer, acquiring vitamin D via sunlight should be undertaken with caution. Moreover, only a few foods, such as fatty fish, fortified milk, eggs, and mushrooms, contain substantial amounts of vitamin D. Therefore, direct supplementation with vitamin D_3_ (800–4000 IU, i.e., 20−100 μg/day) is often needed to boost serum 25(OH)D levels to 30–60 ng/mL (75–150 nmol/L) [45]. It was shown that oral intake of 25(OH)D_3_ (calcifediol) has advantages over vitamin D_3_, and vitamin D_3_ is more effective than vitamin D_2_ in increasing serum levels of 25(OH)D [46,47,48,49,50]. Generally, picomolar concentrations of 1, 25(OH)_2_D_3_ are sufficient to maintain calcium–phosphate homeostasis. However, for cancer prevention or treatment, higher dosages of vitamin D are required, which may increase the risk of hypercalcemia. Notably, 1, 25(OH)_2_D_3_ is unstable and easily converted into its inactive forms by CYP24A1, thus limiting the efficacy of 1, 25(OH)_2_D_3_ as a therapeutic agent. 

To improve the safety and efficacy of 1, 25(OH)_2_D_3_, many vitamin D analogues were developed and tested in clinical trials [51,52]. Single-nucleotide polymorphisms (SNPs) in the genes related to vitamin D metabolism or in VDR may affect serum 25(OH)D levels or individual responsiveness to vitamin D supplementation and are associated with the development and progression of some diseases [35,53]. 

## 3. Vitamin D and Pancreatic Cancer Risk and Incidence 

Researchers examined the role of sunlight or UVB irradiance and vitamin D in cancer risk and progression in geographical ecological studies; in observational studies related to UVB irradiation, oral vitamin D intake, and serum 25(OH)D concentration; and in randomized controlled trials (RCTs). An early clue linking UVB irradiation to the inverse risk of PC was the finding that the incidence of PC in northern latitudes is 3- to 4-times higher than that in areas closer to the equator [54]. This difference was attributed to sunlight or UVB exposure that triggers vitamin D synthesis in humans. Globally, countries in both hemispheres with lower UVB irradiance have a higher incidence of PC, with some exceptions [55]. Similarly, low solar radiation and low temperature were associated with an increased risk of PC in Japan [56]. 

Consistently, higher intake of vitamin D is associated with a lower risk of PC in prospective studies of cohorts including 46,771 men and 75,427 women with over 16 years of follow-up [9]; this notion was also supported by a study of Harvard cohorts [57]. However, in a prospective, nested case–control study that included alpha-tocopherol, beta-carotene cancer prevention in a cohort of male Finnish smokers, subjects with higher pre-diagnostic serum 25(OH)D concentrations had a significantly higher (three fold) risk of PC compared with those with lower concentrations [12]. There are concerns about the studies from this cohort because the serum 25(OH)D concentrations in the subjects may have changed considerably over the 16.7 years of follow-up. Furthermore, the incidence of colon cancer was positively associated with serum 25(OH)D concentration in the cohort, and many other studies showed an inverse correlation [58,59,60,61,62]. The same group, in a nested case–control study in the Prostate, Lung, Colorectal, and Ovarian Screening Trial (PLCO) cohort [63], did not find a strong positive association between 25(OH)D and PC risk. More recently, a pooled analysis of nested case–control studies with 451 cases and 1167 controls from 5 cohorts supported an inverse association, i.e., higher circulating 25(OH)D levels were associated with a lower risk of PC [64]. One meta-analysis of observational studies that included 25 correlative studies with a total of 1,214,995 subjects found that taking vitamins, particularly vitamin D and vitamin B12, decreased the risk of PC [65], but another meta-analysis found no significant associations between vitamin D intake or plasma 25(OH)D levels and PC risk [66]. Another pooled analysis of vitamin D intake from multiple sources (dietary, supplementary, and total) and risk of PC using data from nine case–control studies from the International Pancreatic Cancer Case–Control Consortium (PanC4) found that the increase in dietary intake of vitamin D was associated with an increase in PC risk [67].

These inconsistent results regarding vitamin D status and risk of PC highlight the need for a causal RCT study of vitamin D supplementation. Given that PC is a relatively rare cancer, no such study has been undertaken, but the effects of vitamin D supplementation on total cancer incidence are still informative. A nationwide, randomized, placebo-controlled trial of vitamin D_3_ (cholecalciferol, 2000 IU/day) and omega-3 fatty acids (1 g/day) for the prevention of cardiovascular disease and cancer among men aged 50 and older and women aged 55 and older was conducted in the United States (VITAL, NCT01169259). Among the 25,871 participants, with a median follow-up of 5.3 years, supplementation of vitamin D did not result in a lower incidence of invasive cancer compared with placebo [68], but it did reduce the incidence of advanced (metastatic or fatal) cancer in the overall cohort [69]. In an RCT for prevention of colorectal adenomas, daily supplementation with vitamin D3 (1000 IU), calcium (1200 mg), or both after removal of colorectal adenomas had no significant effect on reducing the risk of recurrent colorectal adenomas over a period of 3 to 5 years [70]. These results are similar to those in two recent meta-analyses that included the VITAL and other recent vitamin D trials [71]. SNPs in *VDR* gene are reported to influence the efficiency of vitamin D_3_ supplementation for preventing advanced colorectal adenomas in an RCT study [72]. Similarly, several SNPs in vitamin D metabolic and VDR signaling pathways are reported to be associated with PC risk (Table 1). However, most studies were conducted in specific geographical regions or in limited numbers of subjects with specific ethnic backgrounds. Thus, the conclusions are inconclusive among different reports. It is possible that differences in SNPs and other genetic variants may contribute to the variability in the efficacy of vitamin D supplementation such as that in the VITAL study. Further study is needed to identify which individuals may have a net benefit from vitamin D supplementation [71,73,74].

## 4. Vitamin D and Pancreatic Cancer Survival and Mortality 

There is increasing evidence at different levels to support the beneficial effect of vitamin D on PC survival. Several studies investigated the impact of sunlight or UVB exposure on PC mortality. An ecological and multifactorial study examined the mortality rates of Caucasian Americans for 1950–1969 and 1970–1994 and found that UVB exposure was inversely correlated to cancer mortality of 15 cancers including PC [75]. Another study showed that increased exposure to sunlight improved overall survival in patients with PC in Turkey [76]. In another study, vitamin D insufficiency and deficiency were prevalent among patients with pancreatic adenocarcinoma, and a 25(OH)D level of less than 20 ng/mL in PC patients with stage III and IV disease was associated with poor prognosis [77]. 

However, in the Cancer and Leukemia Group B (CALGB) 80,303 cohort study, the baseline 25(OH)D levels in patients with advanced PC receiving gemcitabine-based chemotherapy were not associated with survival [13], which suggests that vitamin D has a limited impact on prognosis in advanced stage disease. However, this study had significant limitations, including having only a small number of patients who had sufficient 25(OH)D levels, which diminished the power to examine the association between 25(OH)D levels and outcomes. Moreover, baseline 25(OH)D levels in cancer patients may not be representative of levels throughout their illness but rather could reflect inadequate nutrition and/or outdoor activity resulting from the recent onset of illness and/or burden of cancer [13]. 

In another study analyzing PC survival among 493 patients from 5 prospective US cohorts who were diagnosed with PC from 1984 to 2008, the patients who had sufficient pre-diagnostic plasma levels of 25(OH)D had longer overall survival [14]. Similarly, pre-treatment serum vitamin D deficiency was associated with increased inflammatory biomarkers and short overall survival in PC patients in a prospective study [78]. Additionally, a recent meta-analysis also indicated that high plasma 25(OH)D levels were significantly associated with improved survival in PC patients [66]. 

An RCT of vitamin D supplementation is the gold standard by which to determine the causality of vitamin D in cancer survival and mortality, but currently there are no results from such trials in PC. A case report described a female PC patient who errantly took very high doses of vitamin D at 50,000 U daily for a 10-month period, achieving a serum 25(OH)D level of >150 ng/mL, with no appreciable side effects. Although it is uncertain whether it was related to vitamin D supplementation, her disease was stable for 8 months off of conventional treatment [79]. In an updated meta-analysis of 5 RCTs analyzing total cancer mortality, a total of 1591 deaths were recorded during 3–10 years of follow-up. In the intervention group, 54–135 nmol/L of circulating 25(OH)D was attained, and it was found that vitamin D supplementation significantly reduced total cancer mortality [80]. Two additional meta-analyses of RCTs of vitamin D supplementation also support this notion [71,73]. 

Significantly, in combination with genome-wide screening and experimental validation, VDR was identified as a novel determinant of survival in PC patients [81], and the rs2853564 variant in *VDR* interacted with high pre-treatment levels of 25(OH)D and with gemcitabine treatment to confer longer overall survival of PC patients [81] (Table 1).

Collectively, the results from these observational and clinical studies, together with recent laboratory-based evidence [19,20,82,83], provide a rationale for using vitamin D or its analogues as an economical agent in combination with chemo- or immunotherapy for PC treatment. Several clinical trials of vitamin D (or its analogue paricalcitol) alone or in combination with other treatments for PC are ongoing (Table 2).

## 5. Future Directions 

In order to causally define the impact of vitamin D on PC and optimize the beneficial effort of vitamin D for PC treatment, several challenges must be addressed.

First, although clinical evidence has been accumulating, we have not yet obtained definitive evidence that vitamin D supplementation can effectively improve survival of PC patients or reduce the risk of PC in particular populations. Thus, confirmatory RCTs that include more participants with longer follow-up periods are needed [84]. Given that there were contradictory findings regarding the effect of vitamin D on PC risk and survival and that supplementation of calcium and vitamin D, for example, increased the risk of serrated polyps, important precursors of colorectal cancer, in an RCT [12,85], it is important to elucidate the specific role of vitamin D/VDR signaling in the different stages of PC—initiation, progression, maintenance, and metastasis. These results may help to understand the variability in response to vitamin D with respect to the risk, survival, and mortality and to establish the optimal timing and dosage of vitamin D to achieve the largest benefit in the clinical setting. The effects of vitamin D on other tumors must also be clarified. 

Second, there is no established agreement on the adequate serum level of vitamin D needed for prevention or anti-malignancy effects [22]. The results of meta-analyses support achieving circulating levels of 25(OH)D in the range of 54–135 nmol/L to reduce cancer mortality, but some experts argue that the level should be as high as 100–125 nmol/L (40 to 50 ng/mL) [86]. To achieve such a level of 125 nmol/L, oral supplementation of 4000 IU/day of vitamin D or 2000 IU/day plus exposure of 50% of the body surface to sun for 12 min every day is recommended [86].

Both the supplementation and the clinical uses of high doses of vitamin D have been limited because of the potential occurrence of hypercalcemia. However, over the past few years, hundreds of synthetic vitamin D analogues, which do not affect calcium level but still maintain the antiproliferative and immunomodulatory properties of the active form of vitamin D, have been developed [87,88]. Thus far, very few of these analogues have been studied in clinical trials for PC prevention or treatment. Further studies are needed to optimize the dosage of vitamin D and facilitate the application of vitamin D analogues in clinical settings. 

Third, vitamin D and its analogues exert their functions through binding VDR to regulate downstream target gene expression. Clinical trials of vitamin D or its analogues as therapeutic agents for resectable PC should not only use serum levels of vitamin D as a biomarker but also take into account information about the histopathological characteristics of PC. For example, the status of tumor differentiation, severity of desmoplastic reaction, levels of VDR and CYP24A1 expression, and the status of p53 mutation and VDR SNPs should also be considered in the experimental design and data interpretation, since these factors were reported to affect vitamin D/VDR signaling and treatment response [19,74,81,84,89,90]. Accurately recording these important parameters will allow vitamin D-centered therapies to be applied with greater precision. 

Fourth, immune therapy represents a paradigm shift in cancer treatment, but its efficacy for PC has been largely disappointing [91,92]. With the demonstration that VDR mediates the profound inhibition of pancreatic stellate cell activation and desmoplastic reaction, thereby enabling therapeutic response [20], it seems likely that among the multiplicity of actions of vitamin D, the immunomodulatory effects of vitamin D on both innate and adaptive immunity may ultimately be the most advantageous for PC therapies [93]. For example, expression of functional IL-17 receptors on pancreatic intraepithelial neoplasia (PanIN) induced by Kras^G12D^ and infiltration of the pancreatic stroma by IL-17-producing immune cells induce stem cells features of PanIN and pancreatic cancer cells and accelerate PanIN initiation and progression to invasive tumor [94,95]. Elevated IL-17 production triggers and sustains PC immunosuppression by recruiting neutrophils and excluding cytotoxic CD8^+^ T cells from tumors and renders PC resistant to checkpoint immunotherapy [96]. Vitamin D/VDR signaling significantly suppresses Th17 cell differentiation and transcriptionally inhibits IL-17 expression [97,98,99]. Additionally, vitamin D was found to increase tumor-infiltrating CD8^+^ T cells and decrease tumor growth in a breast cancer model [100]. 1,25(OH)_2_D signaling through the VDR significantly decreases the immunosuppressive capability of myeloid-derived suppressor cell tumor infiltration [101], which is one of the reasons for the inefficiency of checkpoint immunotherapy in PC. However, in a recent study, it was shown that the vitamin D analogue calcipotriol reduced T cell-mediated immunity in 2D and 3D cell culture models, while at the same time, it also reduced the tumor supportive activity of cancer-associated fibroblasts [102]. This study reminds us that the biological effects of vitamin D may be diverse on different cell types or stromal components. Therefore, the net effects of vitamin D should be carefully evaluated in future clinical trials.

Immune checkpoint inhibitors (ICIs) can induce severe immune-related adverse events (irAEs), with common gastrointestinal side effects, including diarrhea and colitis, occurring in up to 30% of patients [103]. The vitamin D/VDR pathway is known to play a role in chronic inflammation of the gastrointestinal tract, such as inflammatory bowel disease, by regulating junctional proteins and inflammatory cytokines [104]. More broadly, many in vitro and in vivo studies revealed that vitamin D and its analogues are effective in reducing systemic or local tissue inflammation [105]. A recent report showed that vitamin D intake is associated with a decreased risk of checkpoint inhibitor-induced colitis [23], possibly owing to the inhibition of IL-17 expression [106]. Thus, it is possible that the administration of vitamin D or its analogues may significantly improve the efficacy of checkpoint immunotherapy and mitigate the irAEs in PC.

Fifth, the gut microbiome represents the collective genetic material within microbiota residing in the human intestinal tract. There is increasing evidence suggesting that gut microbiota play an important role in human health, as well as in the natural history of diseases including cancer and in the therapeutic response [107]. The potential mechanisms of these effects include the involvement of microbiota in the metabolism of nutrients and microbiota-immune system interaction and education [108,109]. An analysis of whole-genome and transcriptome sequencing data from The Cancer Genome Atlas of 33 types of cancer found unique microbial signatures in tissue and blood within and between most major types of cancer [110]. In PC, recent studies showed that both human and mouse pancreatic tumors have distinct microbiome landscapes compared with the normal pancreas [111]. For example, microbiome composition in pancreatic ductal adenocarcinoma patients with long-term survival displayed high tumor microbial diversity and immunoactivation, and a tumoral microbiome signature could predict patients’ survival [112]. The gut microbiome can modulate the PC tumor microbiome, indicating crosstalk between the two microbiomes [112]. Ablation of the tumor microbiome with antibodies reshaped the tumor microenvironment, including reducing myeloid-derived suppressor cells, increasing M1 macrophage differentiation and promoting TH1 differentiation of CD4^+^ T cells and CD8^+^ T-cell activation, and protected against the progression of preinvasive and invasive pancreatic ductal adenocarcinoma, whereas transfer of bacteria from PC patients, but not healthy control subjects, reversed this protective effect [111]. Thus, it is clinically important to identify factors that influence the gut microbiome and develop strategies that manipulate the microbiome to improve the efficiency of immune therapy in PC patients [107].

Vitamin D is known to have an antimicrobial function through activation of immune cells to produce antimicrobial peptides [6,113]. VDR is highly expressed in gut epithetical cells, and vitamin D/VDR signaling plays an important role in regulating gut epithelial proliferation and barrier function as well as the gut microbiome [114,115,116,117]. Distinct effects on fecal microbiota were found in a randomized clinical trial of vitamin D supplementation [118], and higher serum 25(OH)D levels were associated with an increase in beneficial bacteria and a decrease in pathogenic bacteria. A dose-dependent increase in bacteria associated with decreased inflammatory bowel disease activity was observed after various doses of oral vitamin D_3_ supplementation [119]. Lack of the *Vdr* gene caused dysbiosis and promoted tumorigenesis in the intestine in a mouse model [116,120]. However, it remains unknown whether and how vitamin D/VDR signaling affects the PC microbiome and PC development and progression. How best to harness vitamin D’s antimicrobial and regulatory effect on gut microbiota to improve therapeutic efficacy in PC deserves further exploration.

Sixth, VDR displays broad tissue expression, including in pancreatic tissue, but its expression level in pancreatic epithelial cells is relatively low compared to that in gut epithelial cells. Over the past few decades, we have learned much about the functional role of vitamin D/VDR signaling in PC experimentally. For example, reduced VDR expression in pancreatic tumor tissue correlates with poor differentiation, tumor progression, and short survival duration of the patients, and treatment of pancreatic cancer cells with vitamin D or its analogues induces cell cycle arrest and apoptosis and suppresses cancer stemness in vitro and tumorigenesis in vivo [17,121]. More significantly, treatment with vitamin D or its analogue calcipotriol induces stromal reprogramming and potentiates PC response to chemoradiotherapy [19,20]. 

Fundamental questions remain regarding vitamin D/VDR signaling in PC: (*1*) How is VDR signaling involved in acinar-to-ductal metaplasia and early PC, given the critical function of vitamin D/VDR in the regulation of cell differentiation? (*2*) What molecular mechanisms underpin the reduction or loss of VDR expression during PC progression [122]? (*3*) What are the specific molecular targets through which vitamin D/VDR signaling exerts its antitumor activity in PC cells? (*4*) How does vitamin D/VDR signaling mediate the cross-talk between tumor cells and stromal cells to shape the tumor microenvironment and influence tumor progression and therapeutic response? With a *Vdr*-knockout mouse model and CRISPR/Cas9 gene editing, as well as the technical advances in gene/mRNA/16S ribosomal sequencing, we should be able to address these questions and obtain definitive evidence to determine whether vitamin D is effective for the prevention or/and treatment of PC and to develop mechanism-based, effective therapeutic strategies to improve the outcomes of PC. 

In summary, vitamin D suppresses the pleiotropic hallmarks of cancer, and despite the current challenges, accumulating evidence supports the rationale of using vitamin D or its analogues as a cost-effective agent for PC intervention. 

## Figures and Tables

**Table 1:** Relationship of SNPs in vitamin D metabolic and VDR signaling pathways with pancreatic cancer.

Protein Name	Gene Symbol	SNP Locus	Alleles	Location	Amino Acid Variant	Relation to Pancreatic Cancer (PC)	Reference PMID
Vitamin D binding protein	GC	rs2282679	T>G	Intron	NA	No significant correlation	26364161, 31467173
		rs4588	G>A/G>T	Exon	T>M/T>K	No significant correlation	
		rs7041	A>C/A>T	Exon	D>E	No significant correlation	
		rs1491711	C>G	Intron	NA	Heterozygote is associated with PC risk	23826131
25-hydroxyvitamin D-1 alpha hydroxylase	CYP27B1	rs10877012	G>C/G>T	5′ promoter	NA	No association with increased risk for the development of PC	
		rs4646536	A>G	Intron	NA	No association with increased risk for the development of PC	
		rs703842	A>C/A>G/A>T	3′ UTR	NA	No significant correlation	25799011
		rs1048691	C>T	3′ UTR	NA	No significant correlation	
25-hydroxyvitamin D 24-hydrolase	CYP24A1	rs2585428	C>T	Intron	NA	Significantly decrease the risk of PC	29254801
		rs6127119	C>T	Intron	NA	TT versus CC genotype is positively associated with PC risk ^a^	23826131
Vitamin D receptor	VDR	rs2228570	A>C/A>G/A>T	Exon	M>R / M>T / M>K	T allele associates with increased PC risk and tumor pathological differentiation ^b^	25616697, 33226370
		rs1544410	C>A/C>G/C>T	Intron	NA	G allele associates with decreased PC risk and TNM classification ^c^	25616697, 32918214
		rs2853564	G>A	Intron	NA	G allele associates with increased overall survival of PC patients ^d^	30107003

^a^ Not statistically significant after adjustment for multiple comparisons; ^b^ Start codon variant resulting in a longer protein product; ^c^ linkage disequilibrium with the poly(A) microsatellite in 3′ UTR; ^d^ G allele affects binding of IRF4 to VDR and VDR transcriptional activity.

**Table 2:** Representative ongoing clinical trials of vitamin D or its analog combined with other agents for pancreatic cancer treatment.

Trial Identifier	Agents	Patient Condition	Patient Number	Study Phase	Design	Status	Location
NCT04617067	Paricalcitol/Gemcitabine/Nab-paclitaxel	Advanced or metastatic PDAC	43	2	Single Group	Recruiting	Ireland
NCT04524702	Paricalcitol/Gemcitabine/Hydroxychloroquine/Nab-paclitaxel	Advanced or metastatic PDAC	21	2	Single Group	Recruiting	United States
NCT04054362	Paricalcitol/Paclitaxel protein bound/Cisplatin/Gemcitabine	Untreated metastatic PDAC	14	2	Non-Randomized	Recruiting	United Kingdom
NCT03883919	Paricalcitol/5-FU/Leucovorin/Liposomal Irinotecan	Advanced PDAC progressed on Gemcitabine-based therapy	20	1	Non-Randomized	Recruiting	United States
NCT03520790	Paricalcitol/Gemcitabine/Nab-paclitaxel	Untreated metastatic PDAC	112	1/2	Randomized	Active, not recruiting	United States
NCT03519308	Paricalcitol/Nivolumab/Nab-Paclitaxel/Gemcitabine	Untreated resectable PDAC	20	1a	Randomized	Recruiting	United States
NCT03415854	Paricalcitol/Cisplatin/Paclitaxel Protein Bound/Gemcitabine	Untreated metastatic PDAC	14	2	Single Group	Active, not recruiting	United States
NCT03331562	Paricalcitol/Pembrolizumab	Metastatic pancreatic cancer	24	2	Randomized	Completed	United States
NCT03138720	Paricalcitol/Paclitaxel protein bound/Gemcitabine/Cisplatin	Resectable, borderline resectable, or locally advanced (unresectable) PDAC	24	2	Single Group	Recruiting	United States
NCT02930902	Paricalcitol/Gemcitabine Hydrochloride/Nab-paclitaxel/Pembrolizumab	Resectable pancreatic cancer	10	1b	Non-Randomized	Active, not recruiting	United States
NCT02754726	Paricalcitol/Nivolumab/Albumin-bound paclitaxel/Cisplatin/Gemcitabine	Untreated metastatic PDAC	10	2	Single Group	Recruiting	United States
NCT03472833	High-dose (4000 IU/day)/Standard-dose (800 IU/day) vitamin D_3_	Pancreatic cancer with vitamin D deficiency	60	3	Randomized	Recruiting	Austria

Note: Pancreatic ductal adenocarcinoma, PDAC.
